# Occupational swine exposure and Hepatitis E virus, *Leptospira, Ascaris suum* seropositivity and MRSA colonization in Austrian veterinarians, 2017–2018—A cross‐sectional study

**DOI:** 10.1111/zph.12633

**Published:** 2019-08-16

**Authors:** Karin Taus, Friedrich Schmoll, Ziad El‐Khatib, Herbert Auer, Heidemarie Holzmann, Stephan Aberle, Shiva Pekard‐Amenitsch, Stefanie Monschein, Tatjana Sattler, Romana Steinparzer, Franz Allerberger, Daniela Schmid

**Affiliations:** ^1^ Austrian Agency for Health and Food Safety (AGES) Vienna Austria; ^2^ European Programme for Intervention Epidemiology Training (EPIET) European Centre for Disease Prevention and Control (ECDC) Stockholm Sweden; ^3^ Austrian Agency for Health and Food Safety (AGES) Mödling Austria; ^4^ Department of Public Health Sciences Karolinska Institutet Stockholm Sweden; ^5^ Institute for Specific Prophylaxis and Tropical medicine, Centre for Pathophysiology, Infectiology and Immunology Medical University of Vienna Vienna Austria; ^6^ Clinical Institute of Virology Medical University of Vienna Vienna Austria; ^7^ Austrian Agency for Health and Food Safety (AGES) Graz Austria; ^8^ Clinic for Ruminants and Swine University Leipzig Leipzig Germany

**Keywords:** *A. suum*, Austria, Hepatitis E, *Leptospira*, livestock, MRSA colonization

## Abstract

We investigated the prevalence of Hepatitis E Virus (HEV), *Leptospira* and *Ascaris suum* (*A. suum*) seropositivity, and of nasal methicillin‐resistant *Staphylococcus aureus* (MRSA) colonization among Austrian practising veterinarians, and assessed the association with occupational swine livestock exposure. The 261 participants completed a questionnaire on demographics, intensity of occupational swine livestock contact and glove use during handling animals and their secretions. Participants' blood samples were tested for HEV, *Leptospira* and *A. suum* seropositivity and nasal swabs cultured for MRSA. We compared swine veterinarians (defined as >3 swine livestock visits/week) to non‐swine veterinarians (≤3 swine livestock visits/week) with regard to the outcomes through calculating prevalence ratio (PR) and 95% confidence interval (CI). Furthermore, the relationship between occupational swine livestock contact and the study outcomes was examined by age (</≥55 years) and glove usage. The prevalence of nasal MRSA colonization was 13.4% (95% CI: 9.3–17.6), of HEV seropositivity 20.8% (95% CI: 15.8–25.7) and *A. suum* seropositivity 44% (95% CI: 37.7–50.2). The highest anti‐leptospiral antibodies titres were 1:200 (*L. hebdomadis*) and 1:100 (*L. autumnalis, L. caicola*) found in three non‐swine veterinarians. Compared to non‐swine veterinarians, swine veterinarians were 1.9 (95% CI: 1.0–3.4) and 1.5 (95%CI: 1.0–2.3) times more likely HEV seropositive and *A. suum* seropositive, respectively, and 4.8 (95%CI: 2.5; 9.3) times more likely nasally colonized with MRSA. Among glove‐using veterinarians, occupational swine contact was no longer a determinant for HEV seropositivity (PR 1.6; 95% CI: 0.8–2.9). Similar was found for *A. suum* seropositivity, which was no longer associated with occupational swine livestock contact in the subgroup of glove using, ≥55‐year‐old veterinarians (PR: 1.07; 95% CI: 0.4–3.3). Our findings indicate that >3 occupational swine livestock visits per week is associated with HEV and *A. suum* seropositivity and nasal MRSA colonization and that glove use may play a putative preventive role in acquiring HEV and *A. suum*. Further analytical epidemiological studies have to prove the causality of these associations.

**Impacts**

- We found a HEV seroprevalence of 20.8% (95% CI: 15.8–25.7), a *A. suum* seroprevalence of 44.0% (95% CI: 37.7–50.2), and a prevalence of nasal MRSA colonization of 13.4% (95% CI: 9.2–17.6) among practicing Austrian veterinarians. Three veterinarians were *Leptospira* seropositive with a titre of 1:200 (*L. hebdomadis*) and 1:100 (*L. autumnalis*, *L. canicola*).
- Swine veterinarians are more likely to be HEV and *A. suum* seropositive, and nasally colonized with MRSA, compared to non‐swine veterinarians.
- Glove use during handling swines and their secretions may play a preventive role in acquiring HEV and *A. suum*.

## 1 INTRODUCTION

Veterinarians are more likely to acquire zoonotic infections compared to the general population (Baker & Gray, 2009; Taylor, Latham, & Woolhouse, 2001). The swine population represents an important potential reservoir for zoonotic viral, bacterial pathogens and helminths (e.g., hepatitis E virus [HEV], methicillin‐resistant *Staphylococcus aureus* [MRSA], *Leptospira* and *Ascaris suum* [*A. suum*]).

The HEV is classified into four human pathogenic genotypes (gt1–4), with gt1 and gt2 exclusively infecting humans (Mushahwar, 2008). Acute HEV infection is usually self‐limiting and probably less than 5% infected, develop symptoms of acute hepatitis. Domestic swine and wild boars are the main animal reservoir for HEV gt3 and gt4 (Lewis, Wichmann, & Duizer, 2010). Berto et al. (2012) found in commercial swine farms in six European countries, other than Austria, a faecal HEV prevalence in growers of 20%–44% and in fatteners of 8%–73%. An increasing number of locally acquired HEV infections in humans, primarily due to gt3, have been reported in Europe (European Association for the Study of the Liver. Electronic address & European Association for the Study of the, 2018; Kamar, Dalton, Abravanel, & Izopet, 2014; Lewis et al., 2010), mainly by zoonotic transmission, in particular, from domestic swine and wild boars or deer (Purcell & Emerson, 2010). This occurs through direct contact with HEV positive swine faeces (Lewis et al., 2010) or consumption of raw meat products, such as liver, from HEV‐infected swine and wild boars (Di Bartolo et al., 2012; Berto et al., 2012). In Austria, the number of yearly reported cases rose from 17 cases in 2014 to 87 in 2017 (Bundesministerium für Arbeit Soziales Gesundheit und Konsumentenschutz, 2018).

Leptospirosis is one of the most common zoonosis worldwide. The manifestation of human infection with *Leptospira* ranges from subclinical infection to severe clinical disease with multi‐organ failure (Weil's disease) and high case fatality rates (Heymann, 2015). Rodents, cattle, horses, sheep, goat and pigs, and unvaccinated dogs as companion animals are considered common reservoirs for *Leptospira* (Bharti et al., 2003). The transmission to humans occurs through contact of non‐intact skin and intact mucous membranes of eyes, nose and mouth with urine, blood or tissue from infected animals or contaminated water (Heymann, 2015). Occupational risk groups are mineworkers, farmers, agriculture workers, sewer workers, slaughterhouse workers, animal caretakers, fish workers, dairy farmers, military personnel and veterinarians. Exposure to *Leptospira* can also occur during recreational activities such as water sports (Haake & Levett, 2015). In Austria, cases of Leptospirosis are rare (Bundesministerium für Arbeit Soziales Gesundheit und Konsumentenschutz, 2018).

*Ascaris suum* is a parasitic nematode that causes ascariasis in swine following faecal–oral transmission of its eggs (Nejsum et al., 2005). *A. suum* is transmitted to humans through direct contact with eggs in swine faeces and swine manure, in water and soil due to fertilization with swine manure. Food‐borne transmission can occur through consumption of raw, unwashed food contaminated with infective *A. suum* eggs or through consumption of raw pork meat (liver) containing *A. suum* larvae (Deutz, 2017). Most human cases of *A. suum* infection tend to be asymptomatic; typical symptomatic presentation is the larva migrans visceralis (VLM) syndrome (Yoshida, Hombu, Wang, & Maruyama, 2016). Serum samples from patients with VLM syndrome in the Netherlands and Austria showed an *A. suum* seroprevalence of 33% and of 13%, respectively (Pinelli, Herremans, Harms, Hoek, & Kortbeek, 2011).

Livestock‐associated (LA‐) MRSA causing human disease was first reported in 2003, when a MRSA strain typically related to swine was isolated from a cohort of 6/23 patients in the Netherlands (de Neeling et al., 2007). This strain belonged to the multilocus sequence type (MLST) 398. Therefore, colonization of swine and calf livestock with LA‐MRSA has been reported in Europe and Northern America (Mroczkowska et al., 2017; Sharma et al., 2016). Swine farmers and swine veterinarians are at increased risk of exposure to LA‐MRSA (Walter et al., 2016). Transmission occurs through physical contact with colonized animals or through inhalation of LA‐MRSA contaminated dust (Schulz et al., 2012).

## 2 MATERIALS AND METHODS

### 2.1 Study design

We conducted a descriptive and analytical cross‐sectional study among practising veterinarians in Austria. The aim of the descriptive study was to estimate the prevalence of HEV, *Leptospira* and *A. suum* seropositivity and of nasal colonization with MRSA among Austrian practicing veterinarians. The aims of the analytical study were to investigate the association of occupational swine livestock exposure with HEV, *Leptospira* and *A. suum* seropositivity and nasal MRSA positivity and to assess the potential effect of glove use on the association of occupational swine livestock exposure with HEV, *Leptospira*, *A. suum* seropositivity and nasal MRSA positivity.

### 2.2 Study population

In 2017, we recruited a convenience sample of Austrian veterinarians at the three largest Austrian veterinary scientific conferences, which are usually attended by the majority of practicing veterinarians (registered at the Austrian veterinarian chamber), including also most of the Austrian swine veterinarians. At each of these three conferences, the study was announced at the beginning of the main lectures. A booth was available during the session breaks to explain the study and recruit study participants. Inclusion criteria were having been residing and practicing in Austria at least since 2016, consenting to participate to the study and to provide a serum sample and nasal swab and no signs of acute infection with HEV, *Leptospira* and *A. suum* and *Staphylococcus aureus*. The study power was retrospectively calculated using OpenEpi (Dean, Sullivan, & Soe, 2013).

### 2.3 Definition of the outcomes of interest and laboratory testing

The outcomes of interest were past history of infection with HEV, *Leptospira* and *A. suum* indicated by seropositivity and a nasal colonization with MRSA. The study nurse collected serum samples and nasal swab from the consenting participants during congress session's breaks and sent these to the respective National Reference Laboratories for HEV, *A. suum*, *Leptospira* and *Staphylococcus aureus* (*S. aureus*). We defined HEV seropositivity as the presence of anti‐HEV IgG in the serum sample, indicating a case of past or chronic HEV infection, detected by use of a qualitative ELISA (ELISA kit for detection of HEV IgG^®^; Fortress Diagnostics Limited). Test sensitivity and specificity are 100% and 86%, as stated by the manufacturer (Al‐Sadeq, Majdalawieh, Mesleh, Abdalla, & Nasrallah, 2018). *A. suum* seropositivity was defined as the detection of anti‐*A. suum* IgG antibodies by using an in‐house *A. suum* immunoblot (As‐IB) based on larval secreted products as antigen, as previously reported (Schneider, Obwaller, & Auer, 2015). Additionally, serum samples were tested for the presence of anti‐leptospiral antibodies using the microscopic agglutination test (MAT). A panel of 16 live cultures of *Leptospira* reference serovars as antigens and a cut‐off MAT titre for seropositivity of ≥1:100 was used. This test was performed in two steps: (a) two doubling dilutions of each serum, 1:25 and 1:50, were used in an initial screening test; and (b) sera, tested positive in the first step, was titrated up to dilutions of 1:1,600. A positive and a negative control were included for each serovar in each test. The end‐point was the highest dilution of serum, at which 50% agglutination occurred. A nasal MRSA colonization was indicated by a positive MRSA nasal swab culture. Additionally, we spa‐typed the recovered MRSA isolates as described elsewhere (Cuny, Layer, Strommenger, & Witte, 2011).

### 2.4 Data collection and definition of exposure factors

Using a self‐administered questionnaire, we obtained information on the occupational swine livestock exposure status accordingly to five categories of contact intensity, which were defined by the weekly average number of swine livestock visits. This categorization was based on former studies (Wright, Jung, Holman, Marano, & McQuiston, 2008) and adapted to personal experiences from Austrian large animal veterinarians. The five categories of contact intensity were as follows: no contact (0 visit), extreme low contact intensity (>0–1 visit/week), low (>1–3 visits/week), moderate (>3–5 visits/week) and high contact intensity (>5 visits/week). In addition, we collected through the self‐administered questionnaire information on demographics (i.e. age, sex, duration of practice) and factors described to be associated with the risk of HEV, *Leptospira* and *A. suum* infection and MRSA colonization (i.e. putative risk factors of the study outcomes) as potential confounding factors or effect modifiers. These factors were as follows: usage of personal protective equipment (defined as use of gloves and facemask during handling with animals and their secretions), occupational slaughterhouse meat inspection, and occupational swine contact abroad, farming or hunting activity and dietary behaviour (consumption of raw innards, vegetarian, vegan diet). We asked for factors explicitly associated with MRSA colonization: chronic skin disease, skin‐soft tissue infections within the previous 6 months, hospital stay more than 3 days within the past 12 months, recent intake of antibiotics and presence of a healthcare worker among household members. HEV relevant information collected were travel history to HEV‐endemic countries and alcohol consumption, and *Leptospira* relevant information such as camping, freshwater sport activity, and past or present military service.

### 2.5 Data analysis

We described the study population by the five categories of swine livestock contact intensity (as defined above: no contact, extreme low, low, moderate, and high contact intensity). We calculated the prevalence of HEV, *Leptospira* and *A. suum* seropositivity and of nasal MRSA colonization as the proportion of positives among the study population, and the 95% confidence intervals (95% CI), using Wald method (Rosner, 2000). We defined a binary exposure variable for occupational swine livestock contact. For this purpose, we compared participants of each exposure subgroup (i.e., high, moderate, low and extreme low swine livestock contact intensity) to those without occupational swine livestock contact, as the reference group, with respect to the study outcomes (HEV, *Leptospira* and *A. suum* seropositivity and nasal MRSA colonization) through calculating the exposure subgroup‐specific prevalence ratios (PR) and 95% CIs by using univariable Poisson regression models (Martinez et al., 2017). A PR with a 95% confidence interval not including 1 was considered as significant measure of association. These exposure subgroups, significantly associated with the study outcomes, were merged into the exposure group. The veterinarians occupationally exposed to swine livestock as defined and were also referred as to swine veterinarians and the unexposed as to non‐swine veterinarians.

First, we calculated the frequency of the putative risk factors for HEV, *Leptospira*, *A. suum* infection and nasal MRSA colonization among the swine veterinarians, compared to the non‐swine veterinarians through calculating proportion differences and their 95% CIs using the STATA –*cs*– command. Second, we tested the association of the occupational swine livestock exposure with the study outcomes (HEV, *Leptospira*, *A. suum* seropositivity and nasal MRSA colonization), and, in addition, the relationship of the putative risk factors with the study outcomes through calculating the prevalence ratios (PR) with their 95% CIs, using univariable Poisson regression models. Third, we analysed the association of occupational swine livestock exposure with the study outcomes by the putative risk factors, which were found to be associated with the study outcomes, in order to identify confounders and effect modifiers. We calculated strata‐specific PRs (95% CI) of the outcomes and tested for homogeneity of the strata‐specific PRs to determine whether these measures of association are significantly different by using the STATA –*csinter*– command. In case of significant difference, then strata‐specific PRs along with their 95% CI were presented, otherwise, we calculated the Mantel‐Haenszel (M‐H) PR as adjusted measure of association and compared it with the crude PR. At least 20% change in the measure after adjusting for the stratifying variable was considered indicative of confounding. Fourth, we calculated the prevalence of the study outcomes across the subgroups of occupational swine livestock contact intensity and tested the significance of a potential dose effect by using chi‐square test for a trend. All data analyses were performed using Stata/SE 13.1.

## 3 RESULTS

### 3.1 Study population

**Figure 1:**
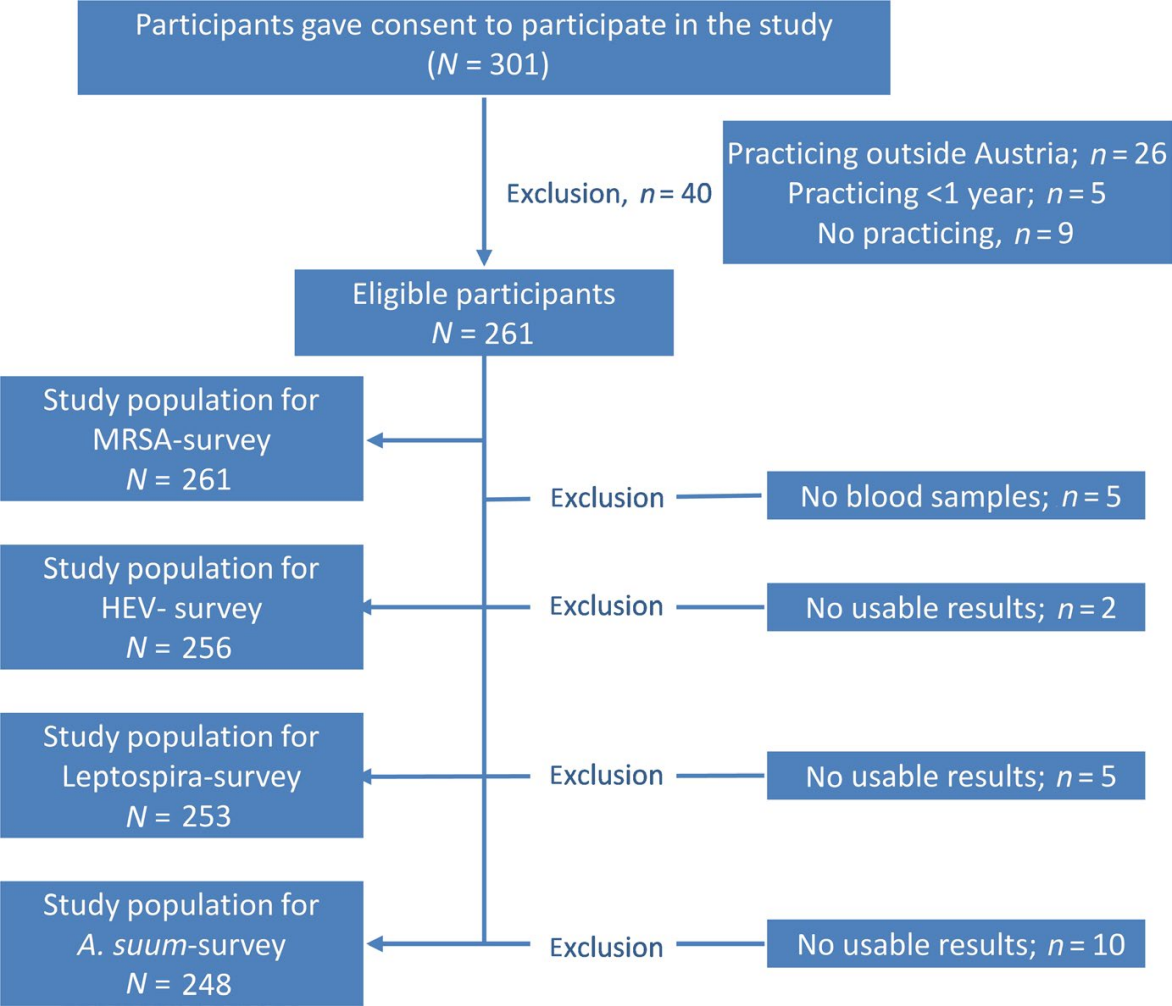
Inclusion and exclusion procedure of the study participants

A total of 301 veterinarians, out of 960 participants at the three conferences (including also non‐ practicing veterinarians, non‐Austrian veterinarians), were initially included in the study. After exclusion of 40 veterinarians (13%) due to practicing outside of Austria (*n* = 26/40), <1 year (*n* = 5/40) or having no direct animal contact (*n* = 9/40), 261 veterinarians remained as eligible study participants. Figure 1 illustrates the inclusion procedure of the study participants for each of the outcomes of interest. A total of 261 participants were tested for MRSA colonization, of these, 256 for HEV seropositivity, 253 for *Leptospira* seropositivity and of these and 248 participants for *A. suum* seropositivity. Considering the sample size of 261 participants including 47 exposed and a prevalence of MRSA colonization of 8% among the unexposed and a significance level of 5% (alpha. 0.05), we were able to identify at least a prevalence ratio of 2.8 with a power of 80%. With a sample size of 256 participants, an unexposed to exposed ratio of 4.4 and a prevalence of HEV seropositivity of 18% among the unexposed, we were able to identify at least a prevalence ratio of 2.1 with a power of 80%. Among the 248 participants including 45 with occupational swine exposure with a prevalence of *A. suum* seropositivity of 40% among the unexposed, we were able to detect at least a prevalence ratio of 1.6 with a power of 80%. Out of the 261 study participants, 173 (66.3%) had no swine livestock contact at all, 21 (8.0%) were allocated to the subgroup of extreme low contact intensity, 20 (7.7%) to the subgroup of low contact intensity, 8 (3.1%) and 39 (14.9%) to the subgroups of moderate and high swine livestock contact intensity, respectively.

### 3.2 Result of the descriptive cross‐sectional study

#### 3.2.1 Prevalence of HEV‐, *Leptospira*‐ and *A. suum* seropositivity and nasal MRSA colonization

Estimates of HEV, *Leptospira* and *A. suum* seroprevalence and nasal MRSA colonization prevalence are given in Table 1. Three participants, all non‐swine veterinarians, were *Leptospira* seropositive: one participant with a single MAT titre of 1:200 against *L. hebdomadis* and two with a single titre of 1:100 against *L. autumnalis* and *L. caicola*, respectively.

**Table 1:** Prevalence of HEV, *Leptospira* and *Ascaris suum* seropositivity and nasal MRSA colonization among the participating veterinarians

Outcomes	Eligible participants (*N*)	Positives (*n*)	%	95% CI
Nasal MRSA colonization	261	35	13.4	9.2; 17.6
HEV seropositivity	256	54	20.8	15.8; 25.7
*Leptospira* seropositivity	253	3	1.2	0.0; 2.5
*A. suum* seropositivity	248	109	44.0	37.7; 50.2

### 3.3 Results of the analytical cross‐sectional study

#### 3.3.1 Defining the binary exposure variable for occupational swine livestock contact

Compared to no occupational livestock contact, the two contact intensity categories >0–1 and >1–3 visits/week showed no association with the study outcomes (HEV, *A. suum* seropositivity, MRSA colonization). Based on these findings (data not shown), we defined the study participants with >3 swine livestock visits/week as occupationally exposed (i.e., swine veterinarians), and participants with ≤3 swine livestock visits/week as unexposed (i.e., non‐swine veterinarians). Accordingly, 47 participants fulfilled the criteria of the exposure group.

#### 3.3.2 Association between occupational swine livestock exposure and other putative risk factors

Table 2 shows the distribution of putative risk factors of the study outcomes, of female sex and older age (>55 years) between the swine veterinarians and the non‐swine veterinarians. Compared to non‐swine veterinarians, there was a significant smaller proportion of non‐user of gloves (−25.9%, 95% CI −40.0; −11.7), of females (−27.0, 95% CI: −41.8; −12.1) and of small animal veterinarians (−27.9, 95% CI: −43.0; −12.8), and a higher proportion of hunting activity (20.3%, 95% CI 6.6; 34.0) and farming (12.3, 95% CI −0.9; 25.6) among the swine veterinarians.

**Table 2:** Frequency distribution of female sex, age ≥55 years, and presence of putative risk factors for the study outcomes between exposed (swine veterinarians) and unexposed (non‐swine veterinarians), *N* = 261, and proportion difference with 95%CI

Other exposure factors	Total (*N* = 261)		Exposed (*n* = 47)		Unexposed (*n* = 214)		Proportion difference	95%CI
	*n*	%	*n*	%	*n*	%	%	
Sex (females)	141	54.0	15	31.9	126	58.9	−27.0	−41.8; −12.1
Age (≥55 years)	45	17.2	10	22.2	35	16.4	4.9	−7.8; 17.6
Small animal veterinarian activity	199	78.0	26	55.3	174	83.2	−27.9	−43.0; −12.8
Presence of chronic skin disease	13	5.0	4	8.5	9	4.2	4.3	−4.2; 12.7
Presence of SSTI in previous 6 months	20	7.7	3	6.4	17	8.0	−1.6	−9.5; 6.3
Hospital stay >3 days, past 12 month	8	3.1	1	2.1	7	3.3	−1.1	−5.9; 3.6
Consumption of antibiotics, previous 7 days	7	2.7	2	4.3	5	2.3	1.9	−4.2; 8.0
Vegetarian diet	11	4.2	1	2.1	10	4.7	−2.6	−7.6; 2.4
Companion animal	232	88.9	41	87.2	191	89.2	−2.0	−12.4; 8.4
Camping activity	62	23.9	8	17.4	54	25.3	−8.0	−20.4; 4.4
Farming activity	40	15.4	12	25.5	28	13.2	12.3	−0.9; 25.6
Hunting activity	34	13.2	14	29.8	20	9.5	20.3	6.6; 34.0
Fresh water sport activity	171	66.5	31	66.0	140	66.7	−0.7%	−15.7; 14.3
Healthcare worker in household members	24	9.3	4	8.9	20	9.4	−5.5	−9.7; 8.7
Travel history to HEV‐endemic countries	191	72.1	37	78.7	154	72.0	6.8	−6.4; 19.9
Non‐use of glove during risky activities	122	46.7	12	25.5	110	51.4	−25.9	−40.0; −11.7
Meat inspection at slaughter house	57	26.0	12	29.3	45	25.3	4.0	−11.3; 19.3
Alcohol consumption ‐ ≥4/week	23	8.7	5	10.6	18	8.4	2.2	−7.3; 11.8

#### 3.3.3 Association of occupational swine livestock exposure and other putative risk factors with the study outcomes

The swine veterinarians were 1.9 (95% CI: 1.0–3.4) and 1.5 (95% CI: 1.0–2.3) times more likely to be HEV and *A. suum* seropositive, respectively and 4.8 (95% CI: 2.5–9.3) times more likely nasally colonized with MRSA, compared to the non‐swine veterinarians (Table 3). We found older age (≥55 years) associated with HEV seropositivity (PR: 2.4, 95% CI: 1.4–4.2) and with *A. suum* seropositivity (PR: 1.5, 95% CI: 1.0–2.3); and non‐glove‐using veterinarians during risky activities (stool sample collection, rectal examination, attendance to birth) 1.4 times more likely to be *A. suum* seropositive than the glove‐using veterinarians (95% CI: 0.9–2.0; Table 3). A Table S1 shows the prevalence ratios (95% CI) of nasal MRSA colonization and HEV seropositivity by their putative risk factors, which were not presented in Table 3. None of these putative risk factors showed an association with MRSA colonization and HEV seropositivity in our study population.

**Table 3:** Prevalence ratio (PR) and 95%CIs of nasal MRSA colonization, and HEV‐ and *Ascaris suum* seropositivity by occupational livestock swine contact as defined (>3 swine livestock visits/week), and by age (≥55 years), sex and non‐use gloves

		Exposed			Unexposed				
Outcome	Participant group	*n*	*N*	%	*n*	*N*	%	PR	95% CI
Nasal MRSA	>3 swine livestock visits/week	18	47	38.3	17	214	7.9	4.8	2.5; 9.3
	Sex (females)	9	141	6.4	26	120	21.7	0.3	0.1; 0.6
	Age (≥55 years)	6	45	13.3	29	216	13.4	1.0	0.4; 2.4
	Non‐use gloves	6	53	11.3	29	208	13.9	0.5	0.3; 1.1
HEV	>3 swine livestock visits/week	16	47	34.0	38	209	18.2	1.9	1.0; 3.4
	Sex (females)	23	138	16.7	31	118	26.3	0.6	0.4; 1.1
	Age (≥55 years)	18	44	40.9	36	212	17.0	2.4	1.4; 4.2
	Non‐use gloves	13	51	25.5	41	209	19.6	0.8	0.5; 1.4
*A. suum*	>3 swine livestock visits/w	27	45	60.0	82	203	40.4	1.5	1.0; 2.3
	Sex (females)	53	133	39.9	56	115	48.7	0.8	0.6; 1.2
	Age (≥55 years)	26	43	60.5	83	205	40.5	1.5	1.0; 2.3
	Non‐use gloves	58	113	51.3	51	135	37.8	1.4	0.9; 2.0

#### 3.3.4 Association of occupational swine livestock exposure with the study outcomes stratified by older age and glove usage

When testing the association of occupational swine contact with HEV, and *A. suum* seropositivity, respectively, by age as the stratifying variable, at the levels, <55 and the ≥55years, we found no evidence of effect modification (test of homogeneity, *p* = .3 for HEV; *p* = .1 for *A. suum*). Among the age group <55 years, the PR of HEV seropositivity among the swine veterinarians, compared with the non‐swine veterinarians was 1.8 (95% CI: 1.0–3.4) and among the age group ≥55 years 1.7 (95% CI: 0.9–3.4). The same holds for the relationship of occupational swine and *A. suum* seropositivity: <55, PR 1.5 (95% CI 1.1; 2.1), ≥55, PR 1.4 (95% CI 0.8; 2.2).

The occupational swine livestock contact was no longer associated with HEV seropositivity among veterinarians using gloves during stool sample collection, rectal examination and attendance to birth (PR 1.6; 95% CI: 0.8–2.9). Among the veterinarians not using gloves, the association remained significant (PR: 2.6; 95% 1.2–5.7). The same holds for *A. suum* seropositivity, for which the association with occupational swine livestock contact remained significant among the non‐glove using ≥55‐year‐old veterinarians (PR: 1.6; 95% CI: 1.1–2.2). We found no effect of glove non‐usage on the association between occupational swine livestock contact and MRSA colonization. The same holds, when we restricted these subgroups analyses to participants with livestock‐associated MRSA colonization. The remaining putative risk factors showed to be neither confounder nor effect modifier for the association between occupational swine exposure and the study outcome.

#### 3.3.5 Dose effect of the occupational swine livestock exposure

The prevalence of nasal MRSA colonization increased with swine contact intensity (>0–1, >1–3 and >3 visits per week), compared to the reference group, no occupational swine exposure. The same holds for the *A. suum* seroprevalence, when using the contact intensities >0–1, >1–5 and >5 swine livestock visits per week, compared to the reference group (Table 4).

**Table 4:** Dose effect: Prevalence of nasal MRSA colonization and *Ascaris suum* seropositivity by decreasing intensity of swine livestock exposure

Pathogen	Exposure categories	*n*	*N*	%	*p*‐Value*
MRSA, *N* = 261	>3 swine livestock visits/week	18	47	38.3	<.01
	>1–3 swine livestock visits/week	2	20	10.0	
	>0–1 swine livestock visits/week	2	21	9.5	
	0 swine livestock visit/week	13	173	7.5	
*A. suum*, *N* = 248	>5 swine livestock visits/week	23	37	62.2	<.01
	>1–5 swine livestock visits/week	15	25	60.0	
	>0–1 swine livestock visits/week	11	21	52.4	
	0 swine livestock visit/week	60	165	36.4	

* *p*‐Value is calculated by using chi‐square test for trend.

## 4 DISCUSSION

This is the first study in Austria estimating the seroprevalence of HEV, *Leptospira* and *A. suum* and the prevalence of nasal colonization with MRSA among veterinarians. We aimed at assessing the association of selected zoonotic diseases with occupational swine livestock contact as we were interested in their potential as occupational diseases among Austrian swine veterinarians.

Our study detected a HEV seropositivity of 21% among all participating veterinarians and of 18% among the non‐swine veterinarians. Previous seroprevalence studies from Austria found among blood donors and soldiers a HEV IgG seropositivity of 13.6% and 14% (Fischer et al., 2015; Lagler et al., 2014), and a recent study in the general population from southern Germany a HEV IgG seroprevalence of almost 18% (Mahrt et al., 2018). We detected an almost two times higher HEV IgG seroprevalence among the swine veterinarians, compared to the non‐swine veterinarians. This is in accordance with findings from the United States and Germany, in which veterinarians and farmers with close and frequent occupational swine contact were 1.5 and 2 times more likely to be HEV seropositive, compared to blood donors (Krumbholz et al., 2012). In our study, veterinarians ≥55 years old were more likely to be HEV seropositive than the <55 years old. This is in accordance with findings from an HEV prevalence study among Finnish veterinarians (Kantala et al., 2016) and may be explained by a higher cumulative occupational risk associated with increasing number of working years. However, older age was neither found as confounder nor as an effect modifier for the association between occupational swine livestock contact and HEV seropositivity. We found, that occupational swine exposure was no longer associated with HEV seropositivity among the veterinarian subgroups, which usually use gloves. But among the veterinarian subgroups working with bare hands, the swine veterinarians were two and a half times more likely HEV seropositive relative to the non‐swine veterinarians.

A *Leptospira* seropositivity, detected only in three non‐swine veterinarians without clinical manifestation, were at low single microagglutination titres (1:200 and 1:100) to *L. hebdomadis*,* L. autumnalis* and *L. caicola*. A cross‐sectional study from the US among 511 veterinarians, found a seroprevalence of 2.5% (Whitney, Ailes, Myers, Saliki, & Berkelman, 2009). In an Austrian study from 1997, 2.9% of 137 veterinarians tested were *Leptospira* seropositive (Nowotny et al., 1997). Two of those had a single high titre of 1:800 to *Leptospira bataviae* and of 1:1,600 to *Leptospira saxkoebing*. Higher *Leptospira* seroprevalence estimates, namely of 10% and 23%, were found in Austrian hunters and in Austrian professional soldiers including military short‐service volunteers. This might be due to more frequent unprotected contact with animal secretions and tissue or contaminated water (Deutz, 2007; Poeppl et al., 2013).

The *A. suum seropositivity* of 44% among all participating veterinarians was surprisingly high, compared to the *A. suum* seroprevalence of 21.9% detected among 137 veterinarians of an Austrian province in a cross‐sectional study (Nowotny et al., 1997). Older age, which we clearly identified as risk factor for *A. suum* seropositivity in our study population, cannot explain this difference, as both study populations showed similar age distribution. We found already a high *A. suum seropositivity* among the non‐swine veterinarians (36% in non‐swine veterinarians). This may be explained by increased consumption of vegetables and fruits in the current study population considering the trend to healthy diet during the last decade. Swine manure is widely used as fertilizer also in vegetable plants in Austria. *A. suum* eggs are extremely resistant to unfavourable environmental conditions and can remain viable up to 10 months in manure, as demonstrated by Katakam, Thamsborg, Kyvsgaard, Dalsgaard, and Mejer (2014). Saying that it was not surprising to find the swine veterinarians only 1.5 times higher *A. suum* seropositive than the non‐swine veterinarians. The probability of *A. suum* seropositivity increased with intensity of occupational swine livestock contact, having found an *A. suum* seroprevalence of 62% among those with more than five swine livestock visits per week. We found glove usage to be, potentially, preventive in the swine veterinarians of older age.

The prevalence of nasal MRSA colonization among the participating veterinarians was with 13% high, compared with a nasal MRSA positivity of 3.8% in 340 veterinarians from Switzerland (Wettstein Rosenkranz et al., 2014) and of 0.2% detected in 3,309 healthy Austrians within a European‐wide cross‐sectional study in 2013 (den Heijer et al., 2013). We found, relative to the non‐swine veterinarians, the swine veterinarians five times more likely to be MRSA positive. The prevalence of nasal MRSA colonization increased with the weekly number of occupational swine livestock visits. Our findings were similar to a study among veterinarians in Germany, which identified a minimum of three weekly visits for swine livestock as the occupational livestock exposure associated with MRSA positivity (Walter et al., 2016). A Dutch prospective cohort study in livestock veterinarians detected that veterinarians with a cumulative duration of at least 3 months of livestock swine contact were at five times higher risk of persistent MRSA carriage, relative to the other livestock veterinarians (Verkade et al., 2013). Wearing a face mask can considerably lower LA‐MRSA carriage in swine farmers (van Cleef et al., 2015). However, we did not find a protective effect of nose‐mouth mask‐use with regard to LA‐MRSA positivity among the swine veterinarians.

### 4.1 Study limitation

The study participants were recruited at the three largest Austrian conferences in 2017, which are usually attended by the majority of the practicing veterinarians in Austria, according to the Austrian Veterinarian chamber (K. Fürwith, personal communication, September 16, 2017). However, a selection bias cannot be excluded as the recruitment among these attendees was on a voluntary basis. Secondly, as the study was underpowered, it has failed to detect associations, in particular, when analysing our primary association by further variables. Third, using seropositivity as an outcome of interest in an analytical cross‐sectional study is prone to the antecedent–consequent bias. Therefore, our findings should be interpreted with caution, but used to generate hypotheses on the veterinary occupational risk of infection with HEV, *A. suum*, *Leptospira*, and of colonization with MRSA.

We detected a low prevalence of *Leptospira* seropositivity among the Austrian veterinarians, compared to previous findings among this occupational group. Our findings indicate that Austrian veterinarians with frequent occupational swine livestock contact are more likely to be HEV and *A. suum* seropositive and nasally colonized with MRSA. Glove use during handling swines and their secretions may play a preventive role in acquiring HEV and *A. suum*. Analytical epidemiological studies have to prove the causality of these findings to make evidence‐based recommendation on glove usage for swine veterinarians.

## CONFLICT OF INTEREST

The authors declare that they have no conflict of interest.

## ETHICAL APPROVAL

The institutional review board of the city of Vienna studied the protocol and decided on 17.01.2017 under EK 17‐003‐VK‐NZ that the study did not require formal ethical review.
