# Supplementary material for: Occupational swine exposure and Hepatitis E virus, Leptospira, Ascaris suum seropositivity and MRSA colonization in Austrian veterinarians, 2017–2018—A cross‐sectional study
Source: Zoonoses Public Health. 2019 Aug 16;66(7):842–51. doi: 10.1111/zph.12633 (PMC6851874; doi:10.1111/zph.12633)
Supplement: Supplementary file 1 [file ZPH-66-842-s001.docx]

**Supplementary table 1. Prevalence ratio (PR) and 95%CIs of nasal MRSA-colonization and HEV seropositivity by their putative risk factors**

|  |  | **Exposed** | | | | | **Unexposed** | | | | | |  | |  |
| --- | --- | --- | --- | --- | --- | --- | --- | --- | --- | --- | --- | --- | --- | --- | --- |
| **Outcome** |  | **n** | | **N** | | **%** | **n** | | **N** | | **%** | | **PR** | | **95% CI** |
| Nasal MRSA | Presence of chronic skin disease | 2 | | 35 | | 5.7 | 11 | | 224 | | 4.9 | | 1.1 | | 0.3; 4.8 |
|  | Presence of SSTI in previous 6 months | 3 | | 35 | | 8.6 | 17 | | 225 | | 7.6 | | 1.1 | | 0.3; 3.7 |
|  | Hospital stay >3 days, past 12 m | 1 | | 35 | | 2.9 | 7 | | 226 | | 3.1 | | 0.9 | | 0.1; 6.8 |
|  | Consumption of antibiotics, previous 7 d | 1 | 35 | | 2.9 | | 6 | 225 | | 2.7 | | 1.1 | | 0.1; 7.8 | |
|  | Health care worker among household members | 4 | 35 | | 11.4 | | 20 | 222 | | 9.0 | | 1.2 | | 0.4; 3.5 | |
| HEV | Travel history to HEV endemic countries | 40 | 189 | | 21.2 | | 14 | 71 | | 19.7 | | 1.1 | | 0.6; 2.0 | |
|  | Hunting activity | 10 | | 34 | | 29.4 | 44 | | 219 | | 20.1 | | 1.5 | | 0.7; 2.9 |
|  | Farming activity | 10 | | 39 | | 25.6 | 44 | | 215 | | 20.5 | | 1.3 | | 0.6; 2.5 |
|  | Alcohol consumption >4 /week | 8 | | 22 | | 36.4 | 46 | | 238 | | 19.3 | | 1.9 | | 0.9; 4.0 |
|  | Meat inspection at slaughter house | 13 | | 54 | | 24.1 | 28 | | 161 | | 17.4 | | 1.4 | | 0.7; 2.7 |
